# Early Maternal Alcohol Consumption Alters Hippocampal DNA Methylation, Gene Expression and Volume in a Mouse Model

**DOI:** 10.1371/journal.pone.0124931

**Published:** 2015-05-13

**Authors:** Heidi Marjonen, Alejandra Sierra, Anna Nyman, Vladimir Rogojin, Olli Gröhn, Anni-Maija Linden, Sampsa Hautaniemi, Nina Kaminen-Ahola

**Affiliations:** 1 Department of Medical Genetics, Faculty of Medicine, University of Helsinki, Helsinki, Finland; 2 Department of Neurobiology, A. I. Virtanen Institute for Molecular Sciences, University of Eastern Finland, Kuopio, Finland; 3 Institute of Biomedicine & Genome-Scale Biology Research Program, Faculty of Medicine, University of Helsinki, Helsinki, Finland; 4 Institute of Biomedicine, Pharmacology, University of Helsinki, Helsinki, Finland; China Agricultural University, CHINA

## Abstract

The adverse effects of alcohol consumption during pregnancy are known, but the molecular events that lead to the phenotypic characteristics are unclear. To unravel the molecular mechanisms, we have used a mouse model of gestational ethanol exposure, which is based on maternal *ad libitum* ingestion of 10% (v/v) ethanol for the first 8 days of gestation (GD 0.5-8.5). Early neurulation takes place by the end of this period, which is equivalent to the developmental stage early in the fourth week post-fertilization in human. During this exposure period, dynamic epigenetic reprogramming takes place and the embryo is vulnerable to the effects of environmental factors. Thus, we hypothesize that early ethanol exposure disrupts the epigenetic reprogramming of the embryo, which leads to alterations in gene regulation and life-long changes in brain structure and function. Genome-wide analysis of gene expression in the mouse hippocampus revealed altered expression of 23 genes and three miRNAs in ethanol-exposed, adolescent offspring at postnatal day (P) 28. We confirmed this result by using two other tissues, where three candidate genes are known to express actively. Interestingly, we found a similar trend of upregulated gene expression in bone marrow and main olfactory epithelium. In addition, we observed altered DNA methylation in the CpG islands upstream of the candidate genes in the hippocampus. Our MRI study revealed asymmetry of brain structures in ethanol-exposed adult offspring (P60): we detected ethanol-induced enlargement of the left hippocampus and decreased volume of the left olfactory bulb. Our study indicates that ethanol exposure in early gestation can cause changes in DNA methylation, gene expression, and brain structure of offspring. Furthermore, the results support our hypothesis of early epigenetic origin of alcohol-induced disorders: changes in gene regulation may have already taken place in embryonic stem cells and therefore can be seen in different tissue types later in life.

## Introduction

Exposure to an adverse environment during pregnancy can harm the developing fetus and have life-long effects on the individual’s health and wellbeing. Maternal alcohol consumption during pregnancy is a leading cause of nongenetic mental retardation and birth defects in the Western world [1], [2]. It can produce fetal alcohol spectrum disorders (FASD), which is an umbrella term for all alcohol-related neurodevelopmental disorders and birth defects. Fetal alcohol syndrome (FAS) with growth restriction, craniofacial dysmorphology, and central nervous system defects represents the most severe end of the FASD continuum. There are several factors contributing to the complex phenotype of alcohol-induced disorders, such as genetic susceptibility, drinking pattern, amount of alcohol, and timing of drinking [3]. Alcohol consumption during early embryogenesis, particularly the time frame around gastrulation when pregnancy may be unknown, has been shown to lead to a high FAS incidence [4], [5], [6].

There is growing evidence to support that the epigenome mediates gene-environment interactions [7], but the molecular mechanisms linking disorders and early life events are unclear. Previous animal studies have shown that ethanol exposure during embryonic development can affect gene expression via epigenetic modifications, such as DNA methylation [8], [9], [10], [11] and non-coding RNAs [11]. The beginning of embryonic development is a period of high DNA synthetic rate and dynamic epigenetic reprogramming [12], [13]. This period appears to be particularly vulnerable to the effects of environmental factors [9], [14], [15], [16], [17], [18] and disruption of these processes can have long-term effects on development [9], [19], [20].

We hypothesize that early gestational ethanol exposure alters the epigenetic reprogramming of the embryo, which leads to alterations in gene regulation and embryonic development, and causes life-long changes in brain structure, function, and behaviour. Previously, we have developed a mouse model of early gestational ethanol exposure, based on maternal *ad libitum* ingestion of 10% (v/v) ethanol between gestational days 0.5–8.5 [9]. This period encompasses pre-implantation, implantation, gastrulation, and the beginning of neurulation. This exposure is considered moderate and chronic, and the exposure period is developmentally equivalent to the first three-four weeks of human pregnancy (clinical gestation age from week three to the beginning of week six). To keep maternal stress as low as possible, we have used mouse strain C57BL/6, which has a strong drinking preference for 10% alcohol [21], [22].

Our previous study demonstrated, for the first time, that ethanol can cause permanent changes to the phenotype of offspring by altering the epigenotype of the early embryo [9]. We discovered that exposure to ethanol increases the DNA-methylation and probability of transcriptional silencing of an epigenetically sensitive allele *Agouti viable yellow* (*A* ^*vy*^) in the offspring. The exposure also caused significant gene expression changes in their liver tissue. The phenotype of the offspring was highly variable, but reminiscent of human FAS with craniofacial dysmorphology and postnatal growth restriction [9], [19]. Increased hyperlocomotion and, unexpectedly, significant improvement in spatial memory in a water maze has also been observed in this mouse model [20].

The aim of this study was to characterize potential changes in the epigenetic regulation of genes in hippocampi caused by early gestational ethanol exposure. The hippocampus is known to be particularly vulnerable to the effects of ethanol. Previous rodent studies have shown that prenatal exposure can reduce the number of hippocampal cells [23], [24], [25], decrease neurogenesis [26], [27], and alter the morphology of neurons [28], [29]. We wanted to see if our exposure is capable of inducing changes in the DNA methylation of other genes along with the epigenetically-sensitive *A* ^*vy*^, leading to altered gene expression. By using a genome-wide gene expression array we found altered expression of 23 genes and three microRNAs in the hippocampi of ethanol-exposed adolescent male offspring at postnatal day (P) 28. We also found site-specific changes in DNA methylation in three CpG islands.

To confirm our array results in hippocampus and to prove our hypothesis of early changes in gene regulation, we tested if similar changes can be detected in gene expression in other tissues. The epigenetic changes in the early embryo that occur prior to cell differentiation are amplified during development by cell divisions, and thus affect numerous cells of different tissue types in the fully grown organism. We selected three candidate genes of which two, *Olfr601* and *H2-M10*.*3*, are known to be expressed actively in the main olfactory epithelium (MOE) and one, *Vpreb2*, in bone marrow. Interestingly, we observed significant ethanol-induced upregulated gene expression in two genes in these three tissues.

This chronic and moderate early gestational ethanol exposure pattern has previously demonstrated distinct phenotypic effects in offspring, but thus far the impact on the structures of the central nervous system remains unclear. To determine the effects on neuronal development that ultimately lead to alterations in offspring brain structure, we performed magnetic resonance imaging (MRI) for adult male offspring (P60) and observed changes in the volumes of hippocampus, olfactory bulb (OB), and ventricles. Most interestingly, we found asymmetry in the volumes of the brain structures: left hippocampus was significantly larger and left OB smaller in the ethanol-exposed offspring.

## Materials and Methods

### Ethic Statement

All the animals were handled and maintained with instructions, orders and ethical principles of EU-directive (European Union). All animal work was approved by the Animal Experiment Board in Finland (ESAVI/3312/04.10.03/2011, ESAVI/976/04.10.07/2013).

### Study design and animals

The mice in this study were inbred, genetically identical, C57BL/6J Rcc (Harlan, Netherlands). The experiments were performed in two animal houses, where all environmental factors (e.g. cage type, environmental enrichment) were standardized for both experimental groups. In total, 18 control (123 offspring, 74 male offspring), 19 ethanol-exposed (137 offspring, 75 male offspring) and 19 cross-fostering control dams were used in this study. Ethanol exposure did not significantly alter litter size (control 6.8±1.6, ethanol-exposed 7.2±1.9, mean±SD, Student’s t-test p = 0.5). The females (8–10 weeks old) were caged with males and the day of plugging was designated gestational day (GD) 0.5. The male was removed from the cage and the water bottle was replaced with a bottle containing 10% (v/v) ethanol. The ethanol solution was changed and consumption was measured every 24 hours. The average daily consumption of 10% ethanol during GD 0.5–8.5 was 3.2±0.6 (mean±SD) ml/mouse/day (or 12g±2.6g ethanol/kg body weight/day). It has been shown that in female mice, consumption of 10% (w/v) ethanol at 14 g ethanol/kg body weight/day produces an average peak blood alcohol level of ≈120mg/dl [30]. A 0.12% blood alcohol level, or approximately 0.10% like in this study, is a realistic human exposure considering that the maximum legal blood alcohol level for driving in Organization for Economic Cooperation and Development (OECD) countries varies from 0.02–0.08% [31]. Pregnant females were allowed free access to the 10% ethanol bottle and food at all time, but water was not available during the exposure period. On the final day of exposure, (GD8.5) the ethanol bottle was replaced with a bottle of tap water. The control females drank tap water through the whole procedure.

A cross-fostering procedure was used to exclude potential alcohol-induced changes in maternal behaviour or care, which could affect the offspring epigenome. The litter from the ethanol-exposed dam was transferred into the cage of the control dam and vice versa within one day of birth. Cross-fostering control offspring were not used as controls in our study. The offspring were left with the dams until weaning at 3 weeks of age. To avoid the potential effects of hierarchy for gene expression in the brain we housed offspring individually for a week. Four-week-old (P28) offspring were sacrificed by cervical dislocation and hippocampi, olfactory bulbs, main olfactory epithelium, and bone marrow from hind-limb bones were dissected. Male mice designated for MRI were housed with male siblings for 5.5 weeks after weaning until anaesthetized and perfused as adults (P57-P60, mainly P60).

### Expression studies

#### Gene Expression Array

For the expression array the hippocampus RNA was extracted by using AllPrep DNA/RNA/Protein Mini Kit and miRNeasy Mini Kit (Qiagen, Valencia, CA, USA). The quality was confirmed by BioAnalyzer (Agilent RNA 600 Nano, Agilent, Germany) and only samples with RNA Integrity Numbers (RINs) above 9 were accepted. The Affymetrix Mouse Exon 1.0 ST Array was used to analyse gene expression in the hippocampi of five control and five ethanol-exposed four-week-old male offspring (from two and three litters, respectively).

The exon array probe expression values were normalized first with the MEAP algorithm [32], [33], [34] followed by background and noise corrections. The normalized probe signals were transformed into gene expression values using MEAP. The analysis was done in the freely available Anduril computational framework [35]. Differential gene expression analysis was performed with statistical significance (Student’s t-test) and fold-change. Genes with nominal p-value less than 0.05 and fold-change (log2-space) more than 1.5 were considered to be differentially expressed.

#### Quantitative real-time PCR

The hippocampus, main olfactory epithelium, and bone marrow RNA for TaqMan procedure was extracted by AllPrep DNA/RNA/Protein Mini Kit or NucleoSpin RNA II kit (hippocampus) (Qiagen, Valencia, CA, USA and Macherey-Nagel, Düren, Germany), Allprep DNA/RNA Mini Kit (MOE) (Qiagen, Valencia, CA, USA), and TRIzol Reagent (bone marrow) (Ambion, Carlsbad, CA, USA). After DNAse treatment (RQ1 RNase-Free DNase, Promega, Madison, WI, USA), cDNA synthesis was performed by using the iScript cDNA Synthesis Kit (BIO-RAD Laboratories, Hercules, CA, USA). TaqMan was performed by using TaqMan Gene Expression Assays (Applied Biosystems, Foster City, CA, USA) and iTaq Universal Probes Supermix kit (BIO-RAD, Laboratories, Hercules, CA, USA). Reaction conditions were as specified by Applied Biosystems. Taqman Assays used for analysis were *Olfr601* (Mm01280848_s1), *H2-M10*.*3* (Mm01277728_g1), and *Vpreb2* (Mm00785621_s1), and housekeeping gene *Rps16* (Mm01617542_g1) as a reference gene for both: MOE and bone marrow. According to our experiments and previous alcohol studies, *Rps16* was a convenient reference gene for this study [36].

**Fig 1:**
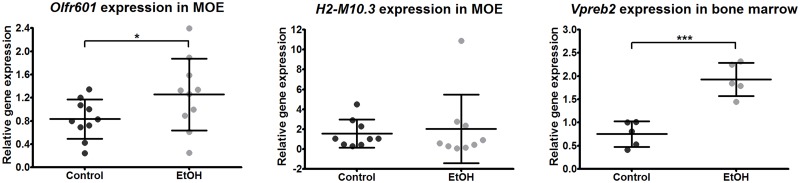
Effects of gestational alcohol exposure on gene expression in main olfactory epithelium and bone marrow. Quantitative PCR studies showed increased expression of *Olfr601* in main olfactory epithelium (MOE) and *Vpreb2* in bone marrow (*p<0.05 and ***p<0.001, respectively, one-tailed Student’s t-test) of ethanol-exposed (EtOH) offspring relative to reference gene *Rps16*. Expression of *H2-M10*.*3* was not significantly changed in MOE (p = 0.35, one-tailed Student’s t-test). Each dot represents an individual four-week-old (P28) male mouse. Bars are averaged values ± SD.

MOEs from 10 control and 10 ethanol-exposed offspring (males from 7 and 5 litters, respectively) were used in TaqMan procedures for *Olfr601* and nine controls and nine ethanol-exposed offspring (males from 5 and 4 litters, respectively) for *H2-M10*.*3* (Fig 1). Five control and five ethanol-exposed offspring (males from 4 and 3 litters, respectively) were used in TaqMan for *Vpreb2* in bone marrow. The qPCR was performed by using the Applied Biosystems 7500 Fast Real Time PCR System (Applied Biosystems, Carlsbad, CA, USA), samples were analysed in triplicates and relative values of expression of genes of interest were determined for each sample using the ΔΔCt method [37]. One tailed Student’s t-test was used to assess differences in relative gene expressions in controls and ethanol-exposed samples.

### Methylation studies

For bisulfite sequencing, hippocampus and MOE samples were extracted using commercial kits (AllPrep DNA/RNA/Protein Mini Kit and Allprep DNA/RNA Mini Kit, Qiagen, Valencia, CA, USA and NucleoSpin RNA II kit, Macherey-Nagel, Düren, Germany) and standard phenol-chloroform protocol. Prior to bisulphite conversion, some hippocampus samples extracted by commercial kits were cleaned by Genomic DNA Clean and Concentrator kit (Zymo Research, Irvine, CA, USA). MOE DNA was cleaned by Proteinase K (Macherey-Nagel, Düren, Germany) and Genomic DNA Clean and Concentrator kit (Zymo Research, Irvine, CA, USA). Sodium bisulphite conversion of hippocampus and main olfactory epithelium DNA was carried out using the EZ methylation kit (Zymo Research, Irvine, CA, USA). For each mouse, one to two bisulphite conversions and 1–2 independent PCR reactions were performed. PCR primers (Sigma-Aldrich, Helsinki, Finland) were designed by using the MethPrim program (The Li Lab, Department of Urology, UCSF) (S1 Table) and the AmpliTaq Gold PCR kit (Applied Biosystems, Carlsbad, CA, USA) was used according to the manufacturer´s protocol. 2 μl of bisulphite treated DNA was used as a template in 25 μl PCR reactions. PCR conditions were: 95°C 10 min; 40 cycles of 95°C 35 s, 56–60°C 35 s (depending on primers), 72°C 1 min; followed by 72°C 10 min. Nested PCR with 0.5 μl -2 μl of template and 35 cycles were used for some PCR fragments (S1 Table). PCR fragments were gel-isolated with NucleoSpin Gel and PCR Clean-up kit (Macherey-Nagel, Düren, Germany) and ligated into the pGEM-T Vector system I (Promega, Madison, WI, USA). Transformation was performed by using standard protocol with 100 μl of DH5α competent cells (Invitrogen, Carlsbad, CA, USA) on ampicillin (100 μg/ml) LB-plates equilibrated with IPTG (Bioline, Taunton, MA, USA) and X-Gal (Promega, Madison, WI, USA). White bacterial colonies were increased in 2 ml of LB and 20 μl of ampicillin (100 μg/ml) overnight. Plasmids were extracted by using NucleoSpin Plasmid and Plasmid EasyPure kits (Macherey-Nagel, Düren, Germany) and sequenced in the Institute for Molecular Medicine Finland (Helsinki, Finland).

**Fig 2:**
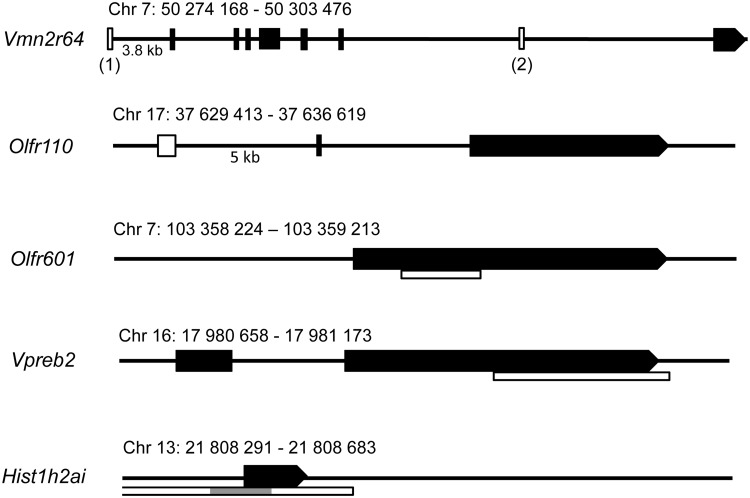
Schematic structures of five candidate genes with six CpG islands. Five candidate genes with six CpG islands were chosen for bisulphite sequencing: *Vomeronasal type 2 receptor 64 (Vmn2r64*, 2 CpG islands (1 and 2), *Olfactory receptor genes 110 (Olfr110)* and *601 (Olfr601)*, *Pre-B lymphocyte gene 2 (Vpreb2)*, *and Histone cluster 1 H2ai (Hist1h2ai)*. Exons are illustrated in black and CpG islands in white. The bisulphite sequenced part of CpG island of *Hist1h2ai* is illustrated in grey.

**Fig 3:**
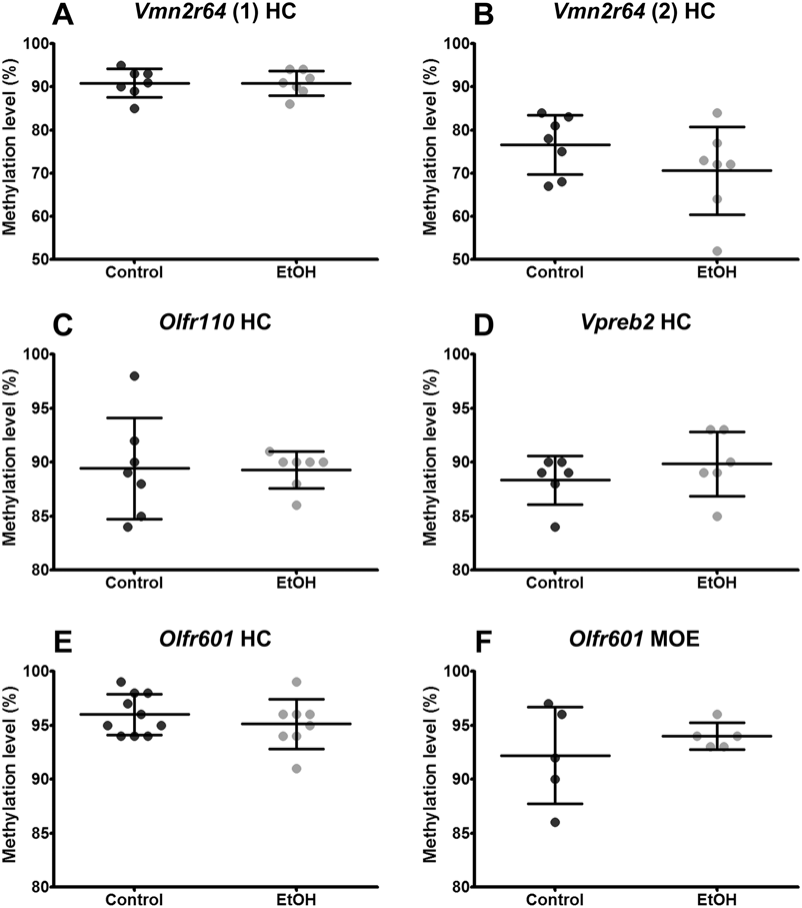
DNA methylation levels of CpG islands of five candidate genes in control and ethanol-exposed offspring. DNA methylation levels of CpG islands in *Vmn2r64* [A: upstream CpG island (1) and B: CpG island in gene-body (2)], *Olfr110* (C, upstream), *Vpreb2* (D, in gene-body), *and Olfr601* (E, in gene-body) in hippocampus (HC) and *Olfr601* (F, in gene-body) in main olfactory epithelium (MOE). Each dot represents an average of methylation percent of clones in a particular CpG island from individual control or ethanol-exposed (EtOH) male offspring (P28).

**Fig 4:**
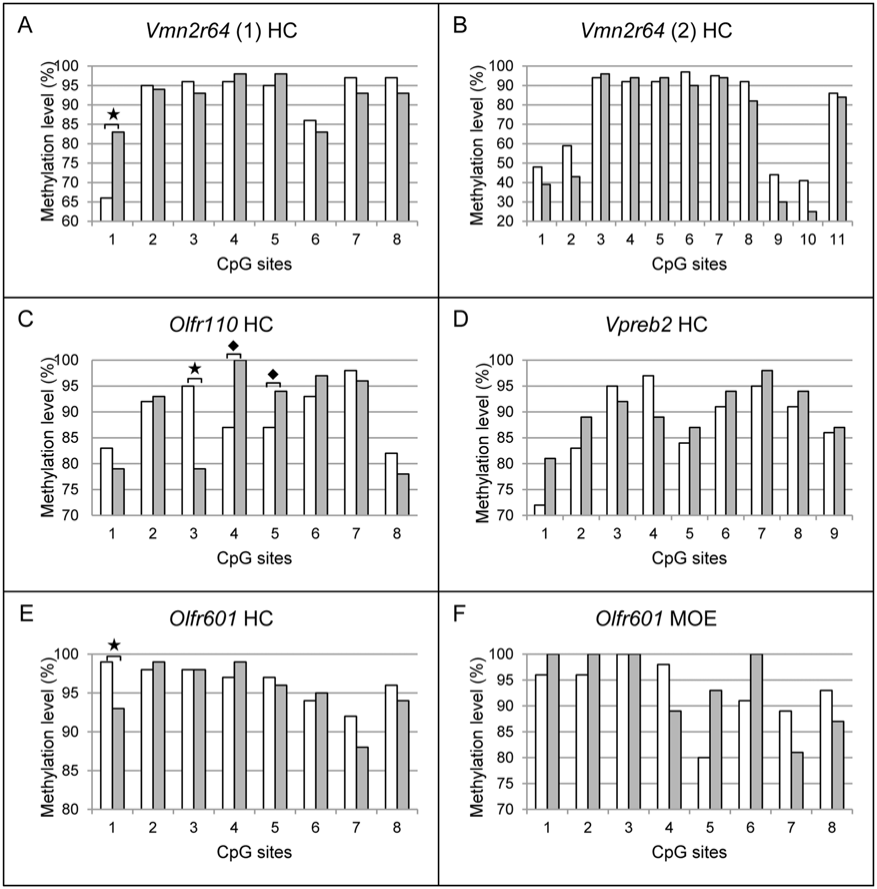
Site-specific DNA methylation levels of six CpG islands in control and ethanol-exposed offspring (P28). Site-specific methylation levels of *Vmn2r64* [A: upstream island (1) and B: CpG island in gene-body (2)], *Olfr110* (C, upstream), *Vpreb2* (D, in gene-body), *and Olfr601* (E, in gene-body) in hippocampus (HC) and *Olfr601* (F, in gene-body) in main olfactory epithelium (MOE). The methylation levels of CpG1 in *Vmn2r64* (1)(A), CpG3 in *Olfr110* (C), and CpG1 in *Olfr601* (E) were significantly changed between the two experimental groups (*p<0.05, Mann-Whitney). Notable changes are marked by diamonds (♦): CpG4 and CpG5 in *Olfr110* (p = 0.07 and p = 0.11, Mann-Whitney, respectively). Controls are illustrated in white and ethanol-exposed offspring in grey bars.

Six CpG islands of five candidate genes differentially expressed in ethanol-exposed offspring in hippocampus expression arrays were bisulphite sequenced: *Olfr110* (7 control and 7 ethanol-exposed offspring, males from 5 and 6 litters, respectively, average amount of clones per mouse was 9), *Olrf601* (10+8 offspring, males from 4 and 4 litters, on average 10 clones/mouse), *Vmn2r64(1)* (8+8 offspring, males from 4 and 4 litters, on average 10 clones/mouse), *Vmn2r64(2)* (7+7 offspring, males from 5 and 6 litters, respectively, on average 9 clones/mouse), *Vpreb2* (6+6 offspring, males from 3 and 4 litters, respectively, on average 9 clones/mouse), and *Hist1h2ai* (6+5 offspring, males from 4 and 3 litters, respectively, on average 7 clones/mouse). *Hist1h2ai* has a long CpG island covering the whole gene-body and promoter region, from which we investigated 43 CpGs (Fig 2). One CpG island of *Olfr601* in MOE (5+5 offspring, males from 3 and 3 litters, on average 9 clones/mouse) was also sequenced (Fig 3F). Sequences were analyzed with BiQ Analyzer [38]. Any clones with lower than 90% conversion rate were excluded from the dataset. The information of CpG-islands was based on the data of the National Center for Biotechnology. Two additional CpG sites for *Vmn2r64(2)* were found (CpG9 and CpG10 in Fig 4B). CpG content in the islands was about 50% or above. Non-parametric Mann-Whitney’s analysis was used to calculate the difference in both, overall DNA methylation of CpG islands and site-specific DNA methylation between two experimental groups.

### Magnetic resonance imaging (MRI)

#### Tissue preparation

Control (n = 8 males from 4 litters) and ethanol-exposed (n = 13 males from 4 litters) mice (P60) were first anaesthetised with an overdose of 100–200 mg/kg pentobarbital (Mebunat, Orion Pharma, Espoo, Finland) i.p. In a deep anaesthesia the mice were transcardially perfused first with 1x phosphate buffered saline (PBS) for 5–10 min followed by 4% paraformaldehyde in 0.1 M PBS, pH 7.4 (5 ml/min, 4°C) for 10 min. The brain was removed from the skull and postfixed in 4% PFA overnight. After this it was washed in 0.9% NaCl for at least 24 h before MRI. Before *ex vivo* MRI, all the brains were immersed in perfluoropolyether (Galden HS240, Ausimont, Milano, Italy) to avoid signal from the solution.

#### Data acquisition and analysis

MRI experiments were carried out in a vertical 9.4 T magnet (Oxford Instruments PLC, Abingdon, UK) interfaced to a Varian DirectDrive console (Varian Inc, Palo Alto, CA) using a quadrature volume RF-coil (diameter 20 mm, Rapid Biomedical GmbH, Rimpar, Germany) for transmitting and receiving. Diffusion tensor imaging (DTI) data were acquired using a diffusion-weighted spin echo sequence (TR = 1.0 s and TE = 26 ms) using 12 diffusion weighting directions with the following parameters: δ = 4.5 ms, Δ = 17 ms and b-value = 1000 s/mm^2^, and one data set without diffusion weighting. The FOV of 20 x 10 x 10 mm^3^ was covered with a 128 x 64 x 64 points. Data were zero padded to 256 x 128 x 128 points resulting in spatial resolution of 78.1 x 78.1 x 78.1 μm^3^. Total scan time was 15 hours.

All data were corrected for eddy current distortions [39], [40] using the FMRIB Software Library (FSL 4.0) software (http://www.fmrib.ox.ac.uk/fsl/). We calculated the diffusion tensor, and the eigenvectors and eigenvalues obtained from the diffusion tensor were used to create maps of fractional anisotropy (FA) [41].

The volumetric analysis was performed on FA maps using in-house built AEDES Matlab software (http://aedes.uef.fi/) (Matlab R2012a). FA maps provided an excellent tissue contrast to outline manually anatomical brain areas selected in this study. We measured total brain volume included cerebrum (olfactory bulbs (OB), basal ganglia, limbic system and cortex), thalamus, midbrain and cerebellum. As selected brain areas, we calculated the volumes of lateral ventricles, hemispheres, hippocampi, olfactory bulbs, and cerebellum. Their volumes were normalized to the total brain volume. Body weight, and whole brain and left/right hemispheres volumes were used as such in the calculations. Cohen´s *d* was calculated to study the effect size between controls and ethanol-exposed mice considering large effect size *d* values ≥0.8 or ≤-0.8 [42]. Positive *d* values indicated increase in volume when negative *d* values showed decrease in volume after ethanol exposure.

## Results

### Early gestational ethanol exposure leads to altered gene expression in adolescent hippocampus, bone marrow and main olfactory epithelium

To detect genome-wide changes in gene expression in hippocampus, we performed expression arrays for four-week-old C57BL/6JRcc male mice from gestational ethanol group (five samples) and controls (five samples). We discovered altered gene expression of 23 genes and three microRNAs (nominal p-values<0.05, FC≥1.5) (Table 1).

**Table 1:** Altered gene expression of 23 genes and three microRNAs in hippocampus of ethanol-exposed offspring (P28).

Upregulated genes:					
Gene	*p*-value	fold change	Gene name	Location	MGI ID
Defb14	0.017	1.7	defensin beta 14	8 A1.3 8	2675345
Olfr937	0.048	1.6	olfactory receptor 937	9 A5	3030771
Cxcr1	0.012	1.6	chemokine (C-X-C motif) receptor 1	1 C3	2448715
H2-M10.3	0.03	1.5	histocompatibility 2, M region locus 10.3	17 B1	1276524
Krtap6-1	0.038	2.1	keratin associated protein 6–1	16 C3.3	1330228
Vpreb2	0.013	1.6	pre-B lymphocyte gene 2	16 A3	98937
Tas2r124	0.006	1.6	taste receptor, type 2, member 124	6 G1	2681267
Olfr601	0.005	1.7	olfactory receptor 601	7 E3	3030435
Mir138-2	0.023	1.5	microRNA 138–2	8 C5	3618733
Gm7168	0.015	3.7	predicted gene 7168	17 A2	3643198
Olfr553	0.01	1.7	olfactory receptor 553	7 E3	3030387
Obox5	0.039	1.6	oocyte specific homeobox 5	7 A2	2149035
Olfr1305	0.0003	1.7	olfactory receptor 1305	2 E5	3031139
Mir290	0.01	1.9	microRNA 290	7 A1	3711323
Mup18	0.022	1.7	major urinary protein 18	4 B3	3705220
Ssxb8	0.014	1.8	synovial sarcoma, X member B, breakpoint 8	X A1.1	2446777
Downregulated genes:					
**Gene**	***p*-value**	**fold change**	**Gene name**	**Location**	**MGI ID**
Olfr967	0.018	0.6	olfactory receptor 967	9 A5.1; 9	3030801
Olfr51	0.012	0.6	olfactory receptor 51	11 B1.3	1333747
Mir16-2	0.032	0.6	microRNA 16–2	3 E1	3618690
C87414	0.041	0.4	expressed sequence C87414	5 E2	2141341
Hist1h2ai	0.023	0.6	histone cluster 1, H2ai	13 A3.1	2448457
Gm904	0.014	0.5	predicted gene 904	13 A5	2685750
Gm15632	0.048	0.5	predicted gene 15632	15 A1	3783076
Krtap27-1	0.004	0.6	keratin associated protein 27–1	16 C3.3	3646229
Olfr110	0.034	0.6	olfactory receptor 110	17 B1	2177493
Vmn2r64	0.032	0.4	vomeronasal type 2 receptor 64	7 B4	3646456

Nominal p-values <0.05, FC ≥ 1.5.

Functionally, the most interesting gene on the array was *Histone cluster 1 H2ai* (*Hist1h2ai*), a linker histone that interacts with linker DNA between nucleosomes, affecting the compaction of chromatin structure. There were also two interesting differentially expressed microRNAs: *miR290* and *miR138-2* (Table 1). *miR290* is associated with gene regulation in the early embryo and the maintenance of the pluripotent cell state [43], [44] and *miR138*, the mature form of *miR138-2*, is associated with the size of dendritic spines in rat hippocampal neurons [45].

The group of genes with altered expression consists of a number of G-protein coupled chemosensory receptors: olfactory receptor genes, which are actively expressed in main olfactory epithelium (*Olfr937*, *Olfr967*, *Olfr51*, *Olfr601*, *Olfr553*, *Olfr1305*, *Olfr110*) and vomeronasal receptor gene (*Vmn2r64)*, which is known to be expressed in the epithelium of the vomeronasal organ. *Taste receptor*, *type 2*, *member 124* (*Tas2r124)* is expressed actively in taste receptor cells in tongue and palate. Three of the up-regulated genes, *defencing beta 14 (Defb14)*, *chemokine (C-X-C motif) receptor 1 (Cxcr1)* and *pre-B lymphocyte gene 2* (*Vpreb2)*, are known to be involved in the function or development of the immune system. *H2-M10*.*3* belongs to the major histocompatibility complex class Ib molecules, but not to the family of classical class I MHC genes, which present antigens to cytotoxic T cells. *H2-M10* genes have been observed to have complex and nonrandom combinations of coexpressions in neurons with *vomeronasal receptor genes* (*Vmn2r*) [46], [47]. A recent study has shown that *H2-M10* genes are required for ultrasensitive chemodetection by a subset of vomeronasal sensory neurons [48].

The expression levels of the differentially expressed genes observed in hippocampus were low and impossible to verify by quantitative PCR (TaqMan). To verify our array results we investigated if similar upregulated expression of *Olfr601* and *H2-M10*.*3* can be seen in both, hippocampus and MOE, and *Vpreb2* in hippocampus and bone marrow. Interestingly, we observed significant upregulated expression in two of the three genes in the tested tissues of ethanol-exposed offspring, *Olfr601* in MOE and *Vpreb2* in bone marrow (p = 0.04 and p<0.001, respectively, one-tailed Student’s t-test) (Fig 1). No significant difference was observed for *H2-M10*.*3* expression between ethanol-exposed and control offspring in MOE (p = 0.35, one-tailed Student’s t-test).

### Altered site-specific DNA methylation in ethanol-exposed offspring

To obtain information about the epigenetic status of the array candidate genes we performed bisulphite sequencing for six CpG islands of five differentially expressed candidate genes. Two of the candidate genes, *Olfr110* and *Vmn2r64*, have CpG islands upstream of the genes and *Vmn2r64*, *Vpreb2*, and *Olfr601* have CpG islands in the gene-body (Fig 2). CpG islands of all the genes except *Hist1h2ai* were highly methylated in both experimental groups (Fig 3). The averages of total methylation levels of all clones were reminiscent and there were no significant differences between ethanol-exposed offspring and controls (Fig 3). There was no difference in DNA methylation between ethanol-exposed and control offspring in *Hist1h2ai* either: in both groups the CpG region that we investigated was hypomethylated.

In addition to overall DNA methylation of CpG islands, we calculated the CpG site-specific DNA methylation in each CpG island (Fig 4). In ethanol-exposed offspring, we observed notable hypermethylation in one CpG site in a CpG island upstream of *Vmn2r64* (CpG1 p = 0.019, non-parametric Mann-Whitney) (Fig 4A), in addition to hypomethylation in one CpG site (CpG3 p = 0.023, Mann-Whitney), and hypermethylation in the next two CpG sites (CpG4 p = 0.07, CpG5 p = 0.11, Mann-Whitney) in an island upstream of *Olfr110* (Fig 4C). The expressions of both genes were downregulated in ethanol-exposed offspring. Furthermore, we found a significantly altered CpG site in exonic CpG island in *Olfr601* (CpG1 p = 0.038, Mann-Whitney) (Fig 4E). This CpG site is hypomethylated and the gene expression is upregulated in hippocampus of ethanol-exposed offspring.

In addition to the hippocampus, we detected significantly upregulated expression of *Olfr601* also in MOE of ethanol-exposed offspring (Fig 1). Due to this, we also sequenced an exonic CpG island of *Olfr601* in MOE to find potential similar changes in DNA methylation caused by gestational ethanol-exposure. We did not observe similar significant ethanol-induced changes in DNA methylation in the hippocampus and MOE, suggesting that this particular exonic CpG site itself does not affect the regulation of *Olfr601* expression (Fig 4F).

### Structural brain abnormalities in ethanol-exposed offspring

**Fig 5:**
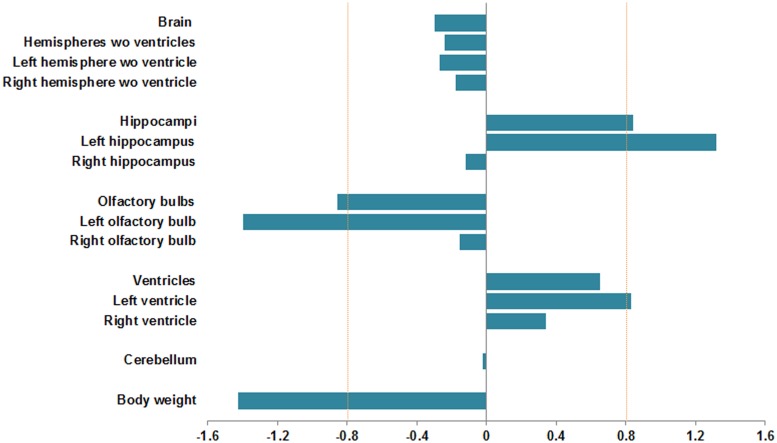
Effect of ethanol exposure on brain regional volumes obtained from MRI images, and body weight. Cohen´s *d* was calculated to study the effect size between controls and ethanol-exposed adult male offspring (P60). Large effect size was considered when *d* values were ≥ 0.8 (increase in volume) or ≤ -0.8 (decrease in volume) after ethanol exposure (dashed line on *d* = ± 0.8).

MRI was performed for 8 control and 13 ethanol-exposed male offspring (P60). The offspring exposed to ethanol showed a reduced body weight as compared to controls (*d* = -1.42) (Fig 5).

We found a small decrease in total brain volume (*d* = -0.29) after ethanol exposure (Fig 5). We measured the effect of ethanol exposure on the size of the hippocampus, OB, ventricles and cerebellum after normalizing their volumes to total brain volume. These regions have been the most severely affected in earlier studies overlapping our ethanol exposure period [42], [49], [50], [51]. There was a large increase in the volume of the hippocampi (*d* = 0.84), slightly increased lateral ventricular volume (*d* = 0.65) and a large decrease in OB (*d* = -0.85) in ethanol-exposed mice (Fig 5). These regions showed asymmetric changes between hemispheres: the left hemisphere exhibited larger changes, such as a large increase in volume in the left hippocampus (*d* = 1.32) and left ventricle (*d* = 0.83) and a large decrease in left OB (*d* = -1.40) (Fig 5). We found a small decrease in the volume of both hemispheres without the contribution of the lateral ventricles (*d* = -0.24), however, individual hemispheres showed slight asymmetric changes (left: *d* = -0.27; right: *d* = -0.18) (Fig 5). No differences were seen in cerebellar volume between controls and ethanol-exposed offspring (*d* = -0.02).

**Fig 6:**
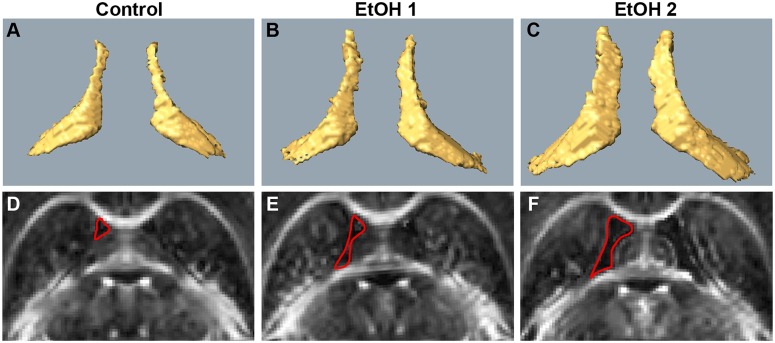
3D visualization of the ventricular volumes of control and ethanol-exposed mice (P60). The top panel shows lateral ventricles of a control (A) and two ethanol-exposed mice (B and C). Fractional anisotropy maps of the same animals are shown in the panel below (D-F). Red line outlines the left ventricle.

**Fig 7:**
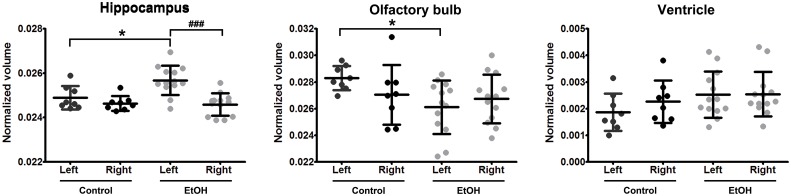
Altered volumes of left and right hippocampi, olfactory bulbs, and lateral ventricles of ethanol-exposed offspring. Wilcoxon test was used to access left-right differences within the same animals (#) and Mann-Whitney test to compare control and ethanol-exposed (EtOH) mice (*). ^#^/*p<0.05 and ^###^p<0.001. Each dot represents an individual adult male mouse (P60). Control mice are illustrated in black and ethanol-exposed offspring in grey. Bars are averaged values ± SD.

Examples of increase in ventricular volume are shown in Fig 6. Control mice showed small lateral ventricular volume as compared to ethanol-exposed mice (Fig 6A and 6D), which varied between animals (Fig 6B,6C and 6E,6F). The most relevant volumetric findings are presented in Fig 7. Left hippocampal volume in ethanol-exposed mice was significantly higher as compared to that in control mice (p<0.05, Mann-Whitney) and to the right hippocampus in the same animals (p<0.001, Wilcoxon test) (Fig 7A). Left olfactory bulb volume was found to be significantly lower as compared to that in control mice (p<0.05, Mann-Whitney) (Fig 7B). The ventricular volumes of both hemispheres appeared slightly larger than those in control mice, however, no significant differences were found (Fig 7C).

## Discussion

### Altered DNA methylation and gene expression in ethanol-exposed offspring

We carried out gene expression arrays for the hippocampus of four-week-old male offspring. According to our hypothesis, alcohol-induced alterations in the epigenome and gene expression in early stage of development are permanent and can be observed later in life. Because the expression profile of a cell and a tissue depends on its function and developmental period, it is challenging to catch primary alterations in gene expressions later in development. Due to that, we took into consideration even very weakly expressed genes on our arrays despite the difficulties to confirm these alterations with other methods.

The microarrays revealed 23 candidate genes and three miRNAs: 15 of the 23 candidate genes belong to gene families that have been associated with gestational ethanol exposure in previous mouse studies. The transcript levels of *Cxcr3*, *Defb15*, *Defb30*, *Krtap4-7*, *Tas2r126*, and *Vmn2r54* have been reported to increase in the whole brain of C57BL/6J adult (P70) male mice that were exposed to maternal 10% ethanol consumption before fertilization, throughout gestation, and ten days after birth (P10) [52]. In addition, *Hist1h3a*, *Hist1h4i*, and *Hist3h2a* were all reported to downregulate in embryos that were cultured and treated with alcohol for 46 hours at early neurulation (E8.25) [53]. Despite different timings and doses of ethanol exposure, the directions of altered expression of our candidate genes (*Cxcr1*, *Defb14*, *Krtap6-1*, *Tas2r124*, *Hist1h2ai*) and genes in the same families mentioned above, were similar. Only in one of the genes, *Vmn2r64*, the direction of change was opposite when compared with its family member *Vmn2r54* in the earlier study [52]. Members of olfactory receptor (Olfr) and keratin associated protein (Krtap) gene families were both up- and downregulated on our array, which suggests that the location and structure of chromatin could determine the effect of ethanol on gene expression. Another whole mouse-embryo culture study also revealed significant changes in DNA methylation in a large number of olfactory receptor genes and decreased methylation in *Hist1h3d* [8], which made olfactory receptor genes and *Hist1h2ai* on our expression array plausible candidate genes for methylation studies. In addition, Liu and colleagues observed a considerable amount of alcohol-induced changes in DNA methylation on chromosome 7 [8], the most common location for our candidate genes: five of our 26 candidate genes and miRNAs are located on this chromosome.

*Hist1h2ai*, the most distinctive candidate gene considering its function, had a lower expression level in ethanol-exposed offspring. We examined only a part of a long CpG island, which was hypomethylated in both experimental groups. The expression arrays revealed seven differentially expressed olfactory receptor genes (*Olfr*s) in hippocampus of ethanol-exposed offspring. *Olfr*s detect volatile chemicals leading to the initial perception of smell in the brain. The members of this large gene family are expressed normally in a monogenic and monoallelic fashion in olfactory sensory neurons in MOE. Their expression is regulated in a specific epigenetic manner, which has been compared to phenomena like genomic imprinting or X-chromosome inactivation. It has been suggested that *Olfr*s are silenced in MOE by hallmarks of constitutive heterochromatin and at a later stage an enzymatic activity removes these hallmarks from a stochastically chosen allele, allowing its transcriptional activity [54]. In addition to MOE, *Olfr*s are known to also express in other tissues, such as brain, but their function remains unclear [55].

We searched for potential changes in DNA methylation of *Olfr110* and *Vmn2r64*. They were both downregulated in our hippocampus array in ethanol-exposed offspring and both have upstream CpG islands. Although the overall methylation level of the CpG islands was similar between the experimental groups, we observed ethanol-induced CpG site-specific alterations in four sites. These upstream regions could have a role in gene regulation, for example as enhancers. Hypermethylated sites in both regulatory regions can disturb the binding of transcription factors and thus slightly suppress gene expression. Although there is earlier evidence of site-specific DNA methylation associating with decreased transcription [56], [57], functional experiments will be needed to clarify whether these alterations in DNA methylation are capable of reducing the transcription of *Vmn2r64* and *Olfr110*.

Expression array results are supported by our TaqMan study. Regardless of the difference between cell types and epigenetic profiles in these three tissues, it was possible to detect significantly upregulated expression in two of the three tested genes. *Olfr601* was upregulated in both the hippocampus and MOE of ethanol-exposed offspring. *Vpreb2* was upregulated in the hippocampus and startlingly overexpressed in bone marrow. *Vpreb2* is one of two mouse *pre-B lymphocyte genes*, *Vpreb1 and Vpreb2*, that are involved in B-cell development. A previous study has shown that the expression of *Vpreb2* is significantly lower compared to *Vpreb1* and it is not needed for the normal development of B cells [58]. Due to the unclear function of *Vpreb2*, further research is needed to clarify if this alteration is caused by alcohol-induced activation of the immune system or dysfunction of epigenetic gene regulation caused by gestational ethanol exposure.

We observed higher methylation in ethanol-exposed offspring in the exonic CpG island of the upregulated *Vpreb2* in the hippocampus. Gene-body DNA hypermethylation has been observed to correlate positively with gene expression levels in previous studies [59], [60]. However, we cannot rule out hydroxymethylation, which has recently been found; especially in embryonic stem cells, adult nervous system, and bone marrow [61], [62]. It has been suggested that hydroxymethylation has a role in DNA demethylation and enhanced gene expression level [63], [64].

### Changes in brain structure caused by gestational alcohol exposure

The most interesting finding concerning brain structure in our mouse model is a trend of asymmetry in hippocampi: enlarged left hippocampus in ethanol-exposed offspring. Our result is similar to a human study, where hippocampal volumes in male adolescents with and without a family history of alcoholism, prior to the initiation of alcohol use of the children themselves, was examined [65]. The participants were typically from upper-middle to upper-class families and they were excluded for any current or past medical, physical, or psychiatric problems [65], [66]. It is tempting to hypothesize that the hippocampal phenotype in this study is primarily caused by early gestational alcohol exposure as in our mouse model. Interestingly, enlargement of hippocampus has been associated with longer-lasting spatial memory in birds [67], [68], and an altered shape of hippocampus with taxi driving in London [69]. Enlarged hippocampus has been observed in autistic children [70], [71] and may also be related to memory function in this disorder [71]. Furthermore, increased hippocampal cell density and improved performance in the Morris water-maze learning paradigm have been detected in the valproic acid rat model of autism [72]. Whether the larger left hippocampus is causing improvement in spatial memory observed in the Morris water-maze in the mouse model we have used in this study [20] requires further research. Although numerous studies with rodents have shown that prenatal ethanol exposure impairs offspring’s spatial memory [73], [74], [75], [76], no effects or even subtle improvement in hippocampal-dependent learning and memory tasks have also been observed [77], [78], [79], [80], [81]. In addition to dose [80], timing of ethanol exposure seems to be significant for the function of the hippocampus: acute ethanol exposure at postnatal day 7 (P7) causes impairment in hippocampal-dependent spatial memory in adult mice, whereas there is no difference between controls and early ethanol-exposed (E8) offspring [81].

We also detected smaller OBs in the ethanol-exposed offspring. Reduced volume of OB in ethanol-exposed offspring (P60), and also impaired odour discrimination, have been observed in a mouse model, in which C57BL/6J dams consume 10% ethanol solution throughout pregnancy [42]. In this model, the exposure was initiated before fertilization and gradually decreased after the birth. They observed a decreased number of neural precursor cells in the subependymal zone of the lateral ventricles, which is the region of adult neural stem cells. They also detected a decreased number of new cells in the OB during the first few postnatal weeks. Our study supports this result and indicates that ethanol exposure only for the first eight days after fertilization is capable of reducing OB volume. Smaller OBs have also been seen in GD17 embryos in a study where pregnant C57BL/6J mice were administered a strong intraperitoneal ethanol dose twice at GD8 [49].

MRI revealed enlargement of lateral ventricles in the ethanol-exposed offspring. Similar results have been seen in MRIs also in other FASD animal models [49], [50], [82], [83], [84], [85] and in clinical studies of prenatal alcohol exposure [86], [87]. Alcohol-induced facial asymmetry [88] and altered symmetry of brain structures [65], [89], [90] have been observed in human studies. In our work, we detected more structural changes in the left side of the brain unlike in the previous MRI study of gestational day 17 (GD17) embryos, whose dams were exposed for acute ethanol insult GD8, in early neurulation stage [49]. They detected the most marked disproportional reduction of regional brain volumes on the right side and the changes were significant in OB, hippocampus, and cerebellum. No difference in the size of cerebellums was observed here, but in both studies, decreased OB volumes and increased ventricular volumes were detected.

In addition to the volumetric analysis, we performed ROI analysis of the dentate gyrus, genu, and body of the corpus callosum and anterior commissure to search microstructural alterations due to the ethanol exposure. No DTI parameters (fractional anisotropy, and axial, radial, and mean diffusivities) showed significant differences when compared to control and ethanol-exposed mice in those areas (data not shown). More detail analysis of DTI data needs further investigation in white and grey matter areas to study the effect of ethanol in tissue microstructure.

## Conclusions

Our study has demonstrated that early, chronic and moderate gestational ethanol exposure affects the development of the embryo and these early changes can be seen in altered DNA methylation, gene expression, and brain structure in later life. The ethanol exposure period in our mouse model developmentally corresponds to the first three-four weeks of human pregnancy, a time period when women are often not aware of their pregnancy. Our results strengthen the significance of environmental factors in early pregnancy and support the role of the epigenome in this interaction.

All the tissues we have studied are regions of actively proliferating stem cells: neuronal progenitor cells in hippocampus, OB (central nervous system) and MOE (peripheral nervous system), and hematopoietic stem cells in bone marrow. This supports our hypothesis of early alcohol-induced epigenetic changes: relatively subtle alterations in the gene regulation could have occurred already in stem cells, affecting both neuronal and hematopoietic cell lines, and can be observed in adult brain structures as well as altered gene expression in brain, bone marrow, and MOE. Although there is an increasing amount of studies that support epigenome involvement in the effects of gestational ethanol exposure, the linkage between early epigenetic changes, altered gene expression, and phenotype characteristics for FASD has not yet been found. In our ongoing work, we aim to reveal the causal epigenetic alterations in early embryonic development and thus increase the understanding of the molecular mechanism behind FASD. Epigenetic changes that alter gene regulation could be considered to be biomarkers and would offer a new tool for the challenging diagnostics of alcohol-induced developmental disorders. Biomarkers in blood, or other available biological samples, would indicate the severity of damage caused by early ethanol exposure. This would enable early diagnosis and appropriate support for development.

## Supporting Information

S1 Table Sequences of primers used in the study. (XLSX)

## References

[1] Abel EL, Hannigan JH. Maternal risk factors in fetal alcohol syndrome: provocative and permissive influences. Neurotoxicol Teratol 17: 445–462. Review. Erratum in: Neurotoxicol Teratol. 1995; 17: 689. 7565491 10.1016/0892-0362(95)98055-6

[2] Sokol RJ, Delaney-Black V, Nordstrom B. Fetal alcohol spectrum disorder. Jama. 2003; 290: 2996–2999. 14665662 10.1001/jama.290.22.2996

[3] Maier SE, West JR. Drinking patterns and alcohol-related birth defects. Alcohol Res Health. 2001; 25: 168–174. 11810954 PMC6707176

[4] Sulik KK. Critical periods for alcohol teratogenesis in mice, with special reference to the gastrulation stage of embryogenesis. Ciba found Symp. 1984; 105: 124–141. 6563984 10.1002/9780470720868.ch8

[5] Guerri C. Mechanisms involved in central nervous system dysfunctions induced by prenatal ethanol exposure. Neurotox. 2002; Res 4: 327–335. 12829422 10.1080/1029842021000010884

[6] Sulik KK. Genesis of alcohol-induced craniofacial dysmorphism. Exp Biol Med (Maywood). 2005; 230: 366–375. 15956766 10.1177/15353702-0323006-04

[7] Feil R, Fraga MF. Epigenetics and the environment: emerging patterns and implications. Nat Rev Genet. 2012; 13: 97–109. 10.1038/nrg3142 22215131

[8] Liu Y, Balaraman Y, Wang G, Nephew KP, Zhou FC. Alcohol exposure alters DNA methylation profiles in mouse embryos at early neurulation. Epigenetics. 2009; 4: 500–511. 20009564 10.4161/epi.4.7.9925 PMC2805036

[9] Kaminen-Ahola N, Ahola A, Maga M, Mallitt KA, Fahey P, Cox TC, et al Maternal ethanol consumption alters the epigenotype and the phenotype of offspring in a mouse model. PLoS Genet 2010; 6: e1000811 10.1371/journal.pgen.1000811 20084100 PMC2797299

[10] Zhou FC, Balaraman Y, Teng M, Liu Y, Singh RP, Nephew KP. Alcohol alters DNA methylation patterns and inhibits neural stem cell differentiation. Alcohol Clin Exp Res. 2011; 35: 735–746. 10.1111/j.1530-0277.2010.01391.x 21223309 PMC3076804

[11] Laufer BI, Mantha K, Kleiber ML, Diehl EJ, Addison SM, Singh SM. Long-lasting alterations to DNA methylation and ncRNAs could underlie the effects of fetal alcohol exposure in mice. Dis Model Mech. 2013; 6: 977–992. 10.1242/dmm.010975 23580197 PMC3701217

[12] Reik W, Dean W, Walter J. Epigenetic reprogramming in mammalian development. Science. 2001; 293: 1089–1093. 11498579 10.1126/science.1063443

[13] Hemberger M, Dean W, Reik W. Epigenetic dynamics of stem cells and cell lineage commitment: digging waddington's canal. Nat Rev Mol Cell Biol. 2009; 10: 526–537. 10.1038/nrm2727 19603040

[14] Morgan HD, Jin XL, Li A, Whitelaw E, O'Neill C. The culture of zygotes to the blastocyst stage changes the postnatal expression of an epigentically labile allele, agouti viable yellow, in mice. Biol Reprod. 2008; 79: 618–623. 10.1095/biolreprod.108.068213 18562706

[15] Rivera RM, Stein P, Weaver JR, Mager J, Schultz RM, Bartolomei MS. Manipulations of mouse embryos prior to implantation result in aberrant expression of imprinted genes on day 9.5 of development. Hum Mol Genet. 2008; 17: 1–14. 17901045 10.1093/hmg/ddm280

[16] Heijmans BT, Tobi EW, Stein AD, Putter H, Blauw GJ, Susser ES, et al Persistent epigenetic differences associated with prenatal exposure to famine in humans. Proc Natl Acad Sci U S A. 2008; 105: 17046–17049. 10.1073/pnas.0806560105 18955703 PMC2579375

[17] Tobi EW, Slagboom PE, van Dongen J, Kremer D, Stein AD, Putter H, et al Prenatal famine and genetic variation are independently and additively associated with DNA methylation at regulatory loci within IGF2/H19. PLoS One. 2012; 7: e37933 10.1371/journal.pone.0037933 22666415 PMC3364289

[18] Susiarjo M, Sasson I, Mesaros C, Bartolomei MS (2013) Bisphenol a exposure disrupts genomic imprinting in the mouse. PLoS Genet 9: e1003401 10.1371/journal.pgen.1003401 23593014 PMC3616904

[19] Kaminen-Ahola N, Ahola A, Flatscher-Bader T, Wilkins SJ, Anderson GJ, Whitelaw E, et al (2010) Postnatal growth restriction and gene expression changes in a mouse model of fetal alcohol syndrome. Birth Defects Res A Clin Mol Teratol 88: 818–826. 10.1002/bdra.20729 20878912

[20] Sanchez Vega MC, Chong S, Burne TH. Early gestational exposure to moderate concentrations of ethanol alters adult behaviour in C57BL/6J mice. Behav Brain Res. 2013; 252: 326–333. 10.1016/j.bbr.2013.06.003 23756143

[21] McClearn G, Rodgers D. Differences in alcohol preference among inbred strains of mice. Q J Stud Alcohol. 1959; 20: 691–695.

[22] Belknap JK, Crabbe JC, Young ER. Voluntary consumption of ethanol in 15 inbred mouse strains. Psychopharmacology (Berl). 1993; 112: 503–510. 7871064 10.1007/BF02244901

[23] Barnes DE, Walker DW. Prenatal ethanol exposure permanently reduces the number of pyramidal neurons in rat hippocampus. Brain Res. 1981; 227: 333–340. 7260643 10.1016/0165-3806(81)90071-7

[24] Diaz Perez H, Espinosa Villanueva J, Machado Salas J. Behavioral and hippocampal morphological changes induced by ethanol administered to pregnant rats. Ann N Y Acad Sci. 1991; 625: 300–4. 2058890 10.1111/j.1749-6632.1991.tb33855.x

[25] Miller MW. Generation of neurons in the rat dentate gyrus and hippocampus: effects of prenatal and postnatal treatment with ethanol. Alcohol Clin Exp Res. 1995; 19: 1500–9. 8749817 10.1111/j.1530-0277.1995.tb01014.x

[26] Choi IY 1, Allan AM, Cunningham LA. Moderate fetal alcohol exposure impairs the neurogenic response to an enriched environment in adult mice. Alcohol Clin Exp Res. 2005; 29: 2053–62. 16340464 10.1097/01.alc.0000187037.02670.59

[27] Gil-Mohapel J, Titterness AK, Patten AR, Taylor S, Ratzlaff A, Ratzlaff T, et al Prenatal ethanol exposure differentially affects hippocampal neurogenesis in the adolescent and aged brain. Neuroscience. 2014; 273: 174–88. 10.1016/j.neuroscience.2014.05.012 24846617

[28] West JR, Hodges CA, Black AC Jr. Prenatal exposure to ethanol alters the organization of hippocampal mossy fibers in rats. Science. 1981; 211: 957–9. 7466371 10.1126/science.7466371

[29] Abel EL, Jacobson S, Sherwin BT. In utero alcohol exposure: functional and structural brain damage. Neurobehav Toxicol Teratol. 1983; 5: 363–6. 6877477

[30] Allan AM, Chynoweth J, Tyler LA, Caldwell KK. A mouse model of prenatal ethanol exposure using a voluntary drinking paradigm. 2003; Alcohol Clin Exp Res 27: 2009–16. 14691390 10.1097/01.ALC.0000100940.95053.72

[31] WHO (World Health Organization) World Health Report 2002: Reducing Risks, Promoting Healthy Life, World Health Organization, Geneva 10.1080/1357628031000116808 14741909

[32] Brazma A, Hingamp P, Quackenbush J, Sherlock G, Spellman P, Stoeckert C, et al Minimum information about a microarray experiment (MIAME)-toward standards for microarray data. Nat Genet. 2001; 29: 365–371. 11726920 10.1038/ng1201-365

[33] Dalma-Weiszhausz DD, Warrington J, Tanimoto EY, Miyada CG. The affymetrix GeneChip platform: an overview. In DNA Microarrays, Part A: Array Platforms and Wet-Bench Protocols, Methods in Enzymology (ed. Kimmel A. and Oliver B.),2006; 410 pp. 3–28. San Diego: Elsevier Academic Press 16938544 10.1016/S0076-6879(06)10001-4

[34] Chen P, Lepikhova T, Hu Y, Monni O, Hautaniemi S. Comprehensive exon array data processing method for quantitative analysis of alternative spliced variants. Nucleic Acids Res. 2011; 39: 123. 10.1093/nar/gkr513 PMC3185423 21745820

[35] Ovaska K, Laakso M, Haapa-Paananen S, Louhimo R, Chen P, Aittomäki V, et al Large-scale data integration framework provides a comprehensive view on glioblastoma multiforme. Genome Med. 2010; 2: 65 10.1186/gm186 20822536 PMC3092116

[36] Carnahan MN, Veazey KJ, Muller D, Tingling JD, Miranda RC, Golding MC. Identification of cell-specific patterns of reference gene stability in quantitative reverse-transcriptase polymerase chain reaction studies of embryonic, placental and neural stem models of prenatal ethanol exposure. Alcohol. 2013; 47: 109–120. 10.1016/j.alcohol.2012.12.003 23317542 PMC3653297

[37] Livak KJ, Schmittgen TD. Analysis of relative gene expression data using real-time quantitative PCR and the 2(-delta delta C(T)) method. Methods. 2001; 25: 402–408. 11846609 10.1006/meth.2001.1262

[38] Bock C, Reither S, Mikeska T, Paulsen M, Walter J, Lengauer T. BiQ analyzer: visualization and quality control for DNA methylation data from bisulfite sequencing. Bioinformatics. 2005; 21: 4067–4068. 16141249 10.1093/bioinformatics/bti652

[39] Jenkinson M, Smith S. A global optimisation method for robust affine registration of brain images. Med Image Anal. 2001; 5: 143–156. 11516708 10.1016/s1361-8415(01)00036-6

[40] Jenkinson M, Bannister P, Brady M, Smith S. Improved optimization for the robust and accurate linear registration and motion correction of brain images. Neuroimage. 2001; 17: 825–841. 10.1016/s1053-8119(02)91132-8 12377157

[41] Basser PJ, Pierpaoli C. Microstructural and physiological features of tissues elucidated by quantitative-diffusion-tensor MRI. J Magn Reson B. 1996; 111: 209–219. 8661285 10.1006/jmrb.1996.0086

[42] Akers KG, Kushner SA, Leslie AT, Clarke L, van der Kooy D, Lerch JP, et al Fetal alcohol exposure leads to abnormal olfactory bulb development and impaired odor discrimination in adult mice. Mol Brain. 2011; 4: 29 10.1186/1756-6606-4-29 21736737 PMC3148973

[43] Houbaviy HB, Murray MF, Sharp PA. Embryonic stem cell-specific MicroRNAs. Dev Cell. 2003; 5: 351–358. 12919684 10.1016/s1534-5807(03)00227-2

[44] Tata PR, Tata NR, Kuhl M, Sirbu IO. Identification of a novel epigenetic regulatory region within the pluripotency associated microRNA cluster, EEmiRC. Nucleic Acids Res. 2011; 39: 3574–3581. 10.1093/nar/gkq1344 21247880 PMC3089473

[45] Siegel G, Obernosterer G, Fiore R, Oehmen M, Bicker S, Christensen M, et al A functional screen implicates microRNA-138-dependent regulation of the depalmitoylation enzyme APT1 in dendritic spine morphogenesis. Nat Cell Biol. 2009; 11: 705–716. 10.1038/ncb1876 19465924 PMC3595613

[46] Ishii T, Hirota J, Mombaerts P. Combinatorial coexpression of neural and immune multigene families in mouse vomeronasal sensory neurons. Curr Biol. 2003; 13: 394–400. 12620187 10.1016/s0960-9822(03)00092-7

[47] Loconto J, Papes F, Chang E, Stowers L, Jones EP, Takada T, et al Functional expression of murine V2R pheromone receptors involves selective association with the M10 and M1 families of MHC class ib molecules. Cell. 2003; 112: 607–618. 12628182 10.1016/s0092-8674(03)00153-3

[48] Leinders-Zufall T, Ishii T, Chamero P, Hendrix P, Oboti L, Schmid A, et al A family of nonclassical class I MHC genes contributes to ultrasensitive chemodetection by mouse vomeronasal sensory neurons. J Neurosci. 2014; 34: 5121–5133. 10.1523/JNEUROSCI.0186-14.2014 24719092 PMC4050176

[49] Parnell SE, O'Leary-Moore SK, Godin EA, Dehart DB, Johnson BW, Allan Johnson G, et al Magnetic resonance microscopy defines ethanol-induced brain abnormalities in prenatal mice: effects of acute insult on gestational day 8. Alcohol Clin Exp Res. 2009; 33: 1001–1011. 10.1111/j.1530-0277.2009.00921.x 19302087 PMC2748865

[50] Godin EA, O'Leary-Moore SK, Khan AA, Parnell SE, Ament JJ, Dehart DB, et al Magnetic resonance microscopy defines ethanol-induced brain abnormalities in prenatal mice: effects of acute insult on gestational day 7. Alcohol Clin Exp Res. 2010; 34: 98–111. 10.1111/j.1530-0277.2009.01071.x 19860813 PMC3506027

[51] Parnell SE, Holloway HE, Baker LK, Styner MA, Sulik KK. Dysmorphogenic effects of first trimester-equivalent ethanol exposure in mice: a magnetic resonance microscopy-based study. Alcohol Clin Exp Res. 2014; 38: 2008–2014. 10.1111/acer.12464 24931007 PMC4107075

[52] Kleiber ML, Laufer BI, Wright E, Diehl EJ, Singh SM. Long-term alterations to the brain transcriptome in a maternal voluntary consumption model of fetal alcohol spectrum disorders. Brain Res. 2012; 1458: 18–33. 10.1016/j.brainres.2012.04.016 22560501

[53] Zhou FC, Zhao Q, Liu Y, Goodlett CR, Liang T, McClintick JN, et al Alteration of gene expression by alcohol exposure at early neurulation. BMC Genomics. 2011; 12: 124 10.1186/1471-2164-12-124 21338521 PMC3056799

[54] Magklara A, Yen A, Colquitt BM, Clowney EJ, Allen W, Markenscoff-Papadimitriou E, et al An epigenetic signature for monoallelic olfactory receptor expression. Cell. 2011; 145: 555–570. 10.1016/j.cell.2011.03.040 21529909 PMC3094500

[55] Kang N, Koo J. Olfactory receptors in non-chemosensory tissues. BMB Rep. 2012; 45: 612–622. 23186999 10.5483/BMBRep.2012.45.11.232 PMC4133803

[56] Martinowich K, Hattori D, Wu H, Fouse S, He F, Hu Y, et al DNA methylation-related chromatin remodeling in activity-dependent BDNF gene regulation. Science. 2003; 302: 890–893. 14593184 10.1126/science.1090842

[57] Jones B, Chen J. Inhibition of IFN-gamma transcription by site-specific methylation during T helper cell development. EMBO J. 2006; 25: 2443–2452. 16724115 10.1038/sj.emboj.7601148 PMC1478170

[58] Mundt C, Licence S, Maxwell G, Melchers F, Martensson IL. Only VpreB1, but not VpreB2, is expressed at levels which allow normal development of B cells. Int Immunol. 2006; 18: 163–172. 16361315 10.1093/intimm/dxh359

[59] Rauch TA, Wu X, Zhong X, Riggs AD, Pfeifer GP. A human B cell methylome at 100-base pair resolution. Proc Natl Acad Sci U S A. 2009; 106: 671–678. 10.1073/pnas.0812399106 19139413 PMC2621253

[60] Aran D, Toperoff G, Rosenberg M, Hellman A. Replication timing-related and gene body-specific methylation of active human genes. Hum Mol Genet. 2011; 20: 670–680. 10.1093/hmg/ddq513 21112978

[61] Kriaucionis S, Heintz N. The nuclear DNA base 5-hydroxymethylcytosine is present in purkinje neurons and the brain. Science. 2009; 324: 929–930. 10.1126/science.1169786 19372393 PMC3263819

[62] Ruzov A, Tsenkina Y, Serio A, Dudnakova T, Fletcher J, Bai Y, et al Lineage-specific distribution of high levels of genomic 5-hydroxymethylcytosine in mammalian development. Cell Res. 2011; 21: 1332–1342. 10.1038/cr.2011.113 21747414 PMC3193467

[63] Tahiliani M, Koh KP, Shen Y, Pastor WA, Bandukwala H, Brudno Y, et al Conversion of 5-methylcytosine to 5-hydroxymethylcytosine in mammalian DNA by MLL partner TET1. Science. 2009; 324: 930–935. 10.1126/science.1170116 19372391 PMC2715015

[64] Guo JU, Su Y, Zhong C, Ming GL, Song H. Hydroxylation of 5-methylcytosine by TET1 promotes active DNA demethylation in the adult brain. Cell. 2011; 145: 423–434. 10.1016/j.cell.2011.03.022 21496894 PMC3088758

[65] Hanson KL, Medina KL, Nagel BJ, Spadoni AD, Gorlick A, Tapert SF. Hippocampal volumes in adolescents with and without a family history of alcoholism. Am J Drug Alcohol Abuse. 2010; 36: 161–167. 10.3109/00952991003736397 20465374 PMC3891832

[66] Nagel BJ, Schweinsburg AD, Phan V, Tapert SF. Reduced hippocampal volume among adolescents with alcohol use disorders without psychiatric comorbidity. Psychiatry Res. 2005; 139: 181–190. 16054344 10.1016/j.pscychresns.2005.05.008 PMC2270700

[67] Clayton NS, Krebs JR. Hippocampal growth and attrition in birds affected by experience. Proc Natl Acad Sci U S A. 1994; 91: 7410–7414. 8052598 10.1073/pnas.91.16.7410 PMC44410

[68] Biegler R, McGregor A, Krebs JR, Healy SD. A larger hippocampus is associated with longer-lasting spatial memory. Proc Natl Acad Sci U S A. 2001; 98: 6941–6944. 11391008 10.1073/pnas.121034798 PMC34457

[69] Maguire EA, Gadian DG, Johnsrude IS, Good CD, Ashburner J, Frackowiak RS, et al Navigation-related structural change in the hippocampi of taxi drivers. Proc Natl Acad Sci U S A. 2000; 97: 4398–4403. 10716738 10.1073/pnas.070039597 PMC18253

[70] Sparks BF, Friedman SD, Shaw DW, Aylward EH, Echelard D, Artru AA, et al Brain structural abnormalities in young children with autism spectrum disorder. Neurology. 2002; 59: 184–192. 12136055 10.1212/wnl.59.2.184

[71] Schumann CM, Hamstra J, Goodlin-Jones BL, Lotspeich LJ, Kwon H, Reiss AL, et al The amygdala is enlarged in children but not adolescents with autism; the hippocampus is enlarged at all ages. J Neurosci. 2004; 24: 6392–6401. 15254095 10.1523/JNEUROSCI.1297-04.2004 PMC6729537

[72] Edalatmanesh MA, Nikfarjam H, Vafaee F, Moghadas M. Increased hippocampal cell density and enhanced spatial memory in the valproic acid rat model of autism. Brain Res. 2013; 1526: 15–25. 10.1016/j.brainres.2013.06.024 23806776

[73] Blanchard BA, Riley EP, Hannigan JH. Deficits on a spatial navigation task following prenatal exposure to ethanol. Neurotoxicol Teratol. 1987; 9:253–258. 3627089 10.1016/0892-0362(87)90010-9

[74] Gianoulakis C. Rats exposed prenatally to alcohol exhibit impairment in spatial navigation test. Behav Brain Res. 1990; 36:217–228. 2310487 10.1016/0166-4328(90)90060-r

[75] Richardson DP, Byrnes ML, Brien JF, Reynolds JN, Dringenberg HC. Impaired acquisition in the water maze and hippocampal long-term potentiation after chronic prenatal ethanol exposure in the guinea-pig. Eur J Neurosci. 2002; 16:1593–1598. 12405973 10.1046/j.1460-9568.2002.02214.x

[76] Christie BR, Swann SE, Fox CJ, Froc D, Lieblich SE, Redila V, et al Voluntary exercise rescues deficits in spatial memory and long-term potentiation in prenatal ethanol-exposed male rats. Eur J Neurosci. 2005; 21: 1719–1726. 15845099 10.1111/j.1460-9568.2005.04004.x

[77] Osborne GL, Caul WF, Fernandez K. Behavioral effects of prenatal ethanol exposure and differential early experience in rats. Pharmacol Biochem Behav. 1980; 12: 393–401. 7393938 10.1016/0091-3057(80)90043-x

[78] Vorhees CV, Fernandez K. Effects of short-term prenatal alcohol exposure on maze, activity, and olfactory orientation performance in rats. Neurobehav Toxicol Teratol. 1986; 8:23–28. 3703092

[79] Clausing P, Ferguson SA, Holson RR, Allen RR, Paule MG. Prenatal ethanol exposure in rats: long-lasting effects on learning. Neurotoxicol Teratol. 1995; 17: 545–552. 8552000 10.1016/0892-0362(95)00014-i

[80] Cullen CL, Burne TH, Lavidis NA, Moritz KM. Low dose prenatal alcohol exposure does not impair spatial learning and memory in two tests in adult and aged rats. PLoS One. 2014; 9: e101482 10.1371/journal.pone.0101482 24978807 PMC4076304

[81] Sadrian B, Lopez-Guzman M, Wilson DA, Saito M. Distinct neurobehavioral dysfunction based on the timing of developmental binge-like alcohol exposure. Neuroscience. 2014; 280: 204–219. 10.1016/j.neuroscience.2014.09.008 25241068 PMC4250396

[82] Mattson SN, Riley EP, Jernigan TL, Garcia A, Kaneko WM, Ehlers CL, et al A decrease in the size of the basal ganglia following prenatal alcohol exposure: a preliminary report. Neurotoxicol Teratol. 1994; 16: 283–289. 7935262 10.1016/0892-0362(94)90050-7

[83] Zhou FC, Sari Y, Powrozek T, Goodlett CR, Li TK. Moderate alcohol exposure compromises neural tube midline development in prenatal brain. Brain Res Dev Brain Res. 2003; 144: 43–55. 12888216 10.1016/s0165-3806(03)00158-5

[84] Sakata-Haga H, Sawada K, Ohnishi T, Fukui Y. Hydrocephalus following prenatal exposure to ethanol. Acta Neuropathol. 2004; 108: 393–398. 15365720 10.1007/s00401-004-0901-8

[85] O'Leary-Moore SK, Parnell SE, Godin EA, Dehart DB, Ament JJ, Khan AA, et al Magnetic resonance microscopy-based analyses of the brains of normal and ethanol-exposed fetal mice. Birth Defects Res A Clin Mol Teratol. 2010; 88:953–964. 10.1002/bdra.20719 20842647 PMC3445267

[86] Johnson VP, Swayze VW II, Sato Y, Andreasen NC. Fetal alcohol syndrome: craniofacial and central nervous system manifestations. Am J Med Genet. 1996; 61: 329–339. 8834044 10.1002/(SICI)1096-8628(19960202)61:4<329::AID-AJMG6>3.0.CO;2-P

[87] Swayze VW 2nd, Johnson VP, Hanson JW, Piven J, Sato Y, Gledd JN, et al Magnetic resonance imaging of brain anomalies in fetal alcohol syndrome. Pediatrics. 1997; 99: 232–240. 9024452 10.1542/peds.99.2.232

[88] Klingenberg CP, Wetherill L, Rogers J, Moore E, Ward R, Autti-Rämö I, et al Prenatal Alcohol Exposure Alters the Patterns of Facial Asymmetry. Alcohol. 2010; 44: 649–657. 10.1016/j.alcohol.2009.10.016 20060678 PMC2891212

[89] Riikonen R, Salonen I, Partanen K and Verho S. Brain perfusion SPECT and MRI in foetal alcohol syndrome. Dev Med Child Neurol. 1999; 41: 652–659. 10587040 10.1017/s0012162299001358

[90] Sowell ER, Thompson PM, Peterson BS, Mattson SN, Welcome SE, Henkenius AL, et al Mapping cortical gray matter asymmetry patterns in adolescents with heavy prenatal alcohol exposure. Neuroimage. 2002; 17: 1807–1819. 12498754 10.1006/nimg.2002.1328

